# Effectiveness of dairy products to protect against cognitive decline in later life: a narrative review

**DOI:** 10.3389/fnut.2024.1366949

**Published:** 2024-06-19

**Authors:** Rachel C. Anderson, Fiona M. Alpass

**Affiliations:** ^1^Te Ohu Rangahau Kai, AgResearch, Palmerston North, New Zealand; ^2^Health and Ageing Research Team, School of Psychology, Massey University, Palmerston North, New Zealand

**Keywords:** dairy, milk, cheese, yogurt, mild cognitive impairment, dementia, Alzheimer's disease

## Abstract

As the world's population ages the prevalence of age-related health concerns is increasing, including neurodegeneration disorders such as mild cognitive impairment, vascular dementia and Alzheimer's disease. Diet is a key modifiable risk factor for the development of neurodegeneration, likely due to gut-brain axis interactions related to neuroinflammation. Analyses of dietary patterns identified dairy as being part of a cognitively healthy diet; however, its contribution to cognitive outcomes is difficult to discern. This narrative review evaluates the literature to determine whether there is sufficient evidence that the consumption of dairy products helps to maintain cognitive function in later life. A search using the terms (dairy OR milk OR cheese OR yogurt OR yogurt) AND (“mild cognitive impairment” OR dementia OR “Alzheimer's disease”) identified 796 articles. After screening and sorting, 23 observational studies and 6 intervention studies were identified. The results of the observational studies implied that the relationship between total dairy consumption and cognitive outcomes is inverse U-shaped, with moderate consumption (1–2 servings per day) being the most beneficial. The analysis of the intake of different types of dairy products indicated that fermented products, particularly cheese, were most likely responsible for the observed benefits. The experimental studies all used dairy-derived peptides produced during fermentation as the dietary intervention, and the results indicated that these could be an effective treatment for early-stage cognitive impairment. Further experimental studies with whole dairy products, particularly fermented dairy, are needed to determine whether the regular consumption of these foods should be recommended to maximize the likelihood of healthy cognitive aging.

## Introduction

The percentage of the world's population that is over 60 years of age is expected to almost double by 2050 (1). Alongside this increase in population age, there will inevitably be a corresponding increase in age-related conditions. Given the changing population dynamics, there will be a marked decrease in the number of working-aged adults compared to those who require aged care, which will put a strain on the medical resources needed to support the health and wellbeing of the aged. Therefore, it is critical to implement preventative measures that increase the likelihood of healthy aging.

Neurodegeneration is one age-related ailment where early intervention to reduce the risk of occurrence may be possible. A decline in cognitive function is a natural part of the aging process (2). However, in some individuals, the decline is greater than that expected during healthy aging. This includes mild cognitive impairment (MCI) where cognitive ability is reduced, typically in verbal episodic memory, but the ability to carry out everyday tasks is retained, as well as vascular dementia and Alzheimer's disease (AD) in which progressive cognitive decline limits the individual's ability to function independently (3).

**Figure 1:**
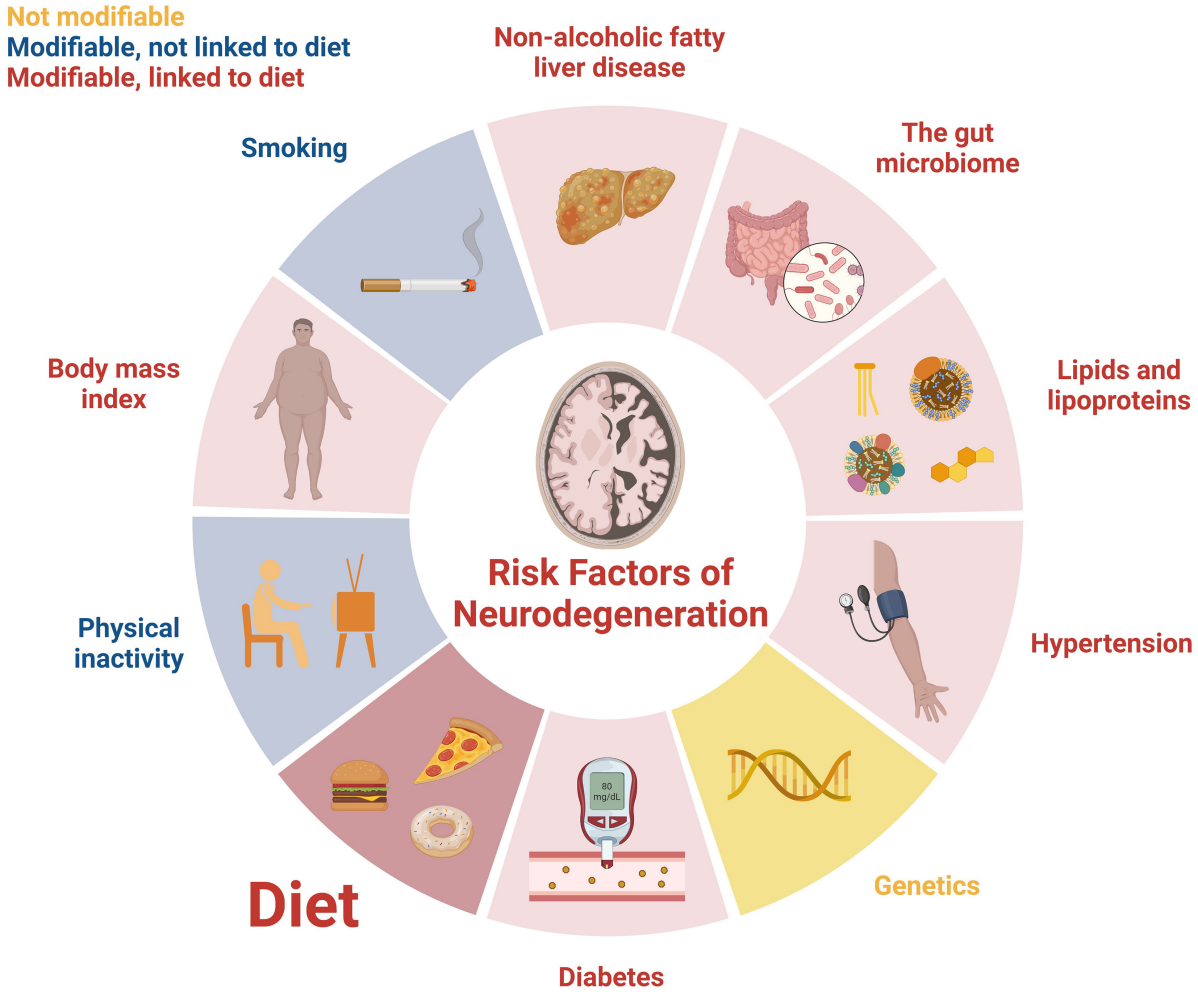
Risk factors for the development of neurodegeneration in later life. Image adapted from a template using BioRender.com.

Diet is a key risk factor for the development of neurodegeneration in later life (Figure 1). Although there are some associations between genetics and neurodegeneration, most of the risk factors for later-life cognitive impairment are modifiable (4). Of the modifiable risk factors, diet is critical because it plays a role in the development of the majority of the other risk factors. This includes those associated with metabolic syndrome (obesity, unhealthy lipid profiles, hypertension, insulin resistance, non-alcoholic fatty liver disease), as well as the gut microbiome composition.

**Figure 2:**
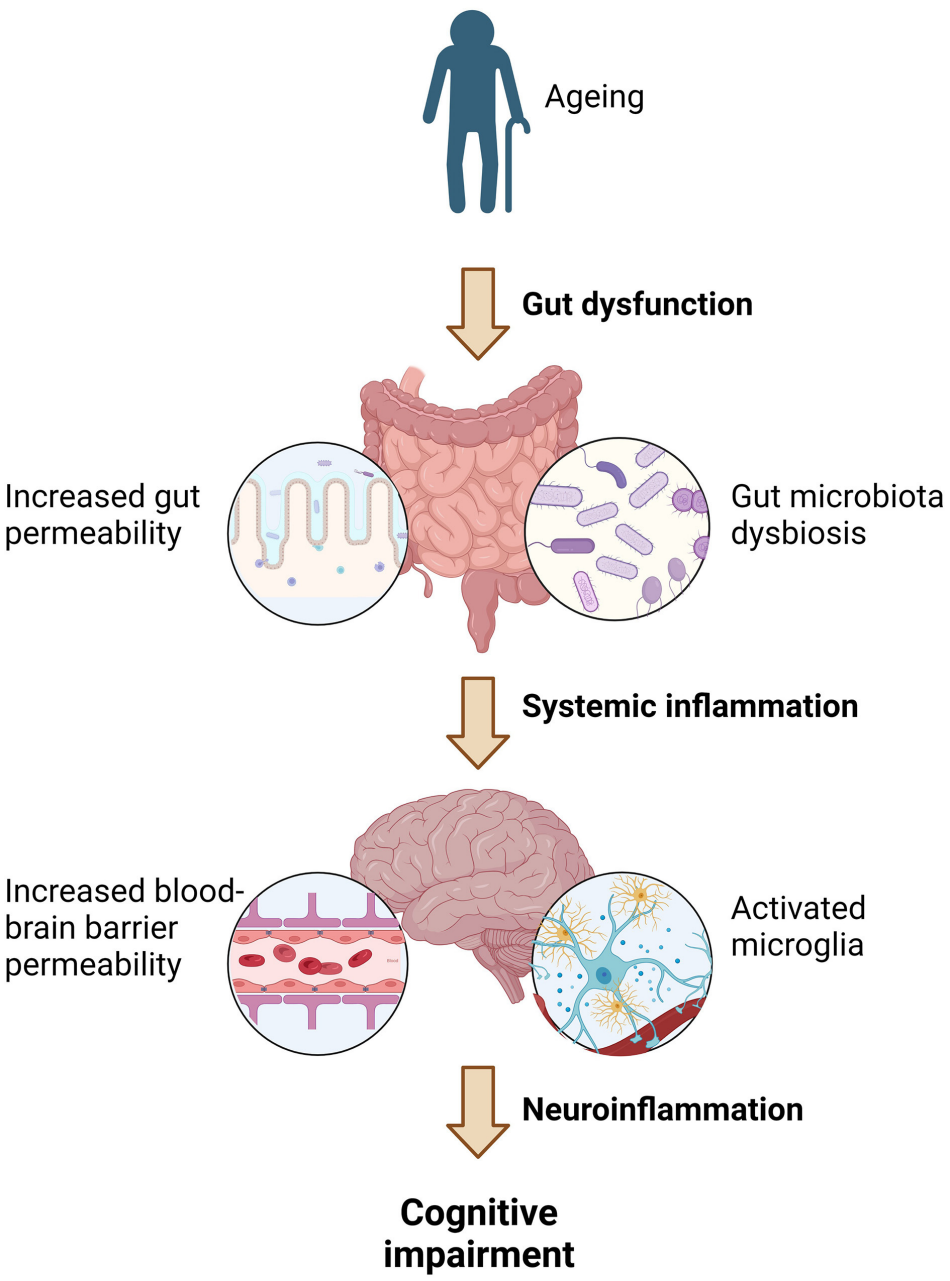
The proposed pathway by which age-associated gut dysfunction may contribute to cognitive impairment. Image created using BioRender.com.

As well as influencing the other risk factors for neurodegeneration, a healthy diet may protect against cognitive impairment by mitigating gut-derived neuroinflammation (Figure 2). During the natural aging process changes in gut function occur (5). This includes alterations in microbiota composition and increased gut permeability which enables antigens to enter the body. This in turn triggers chronic systemic inflammation, which in a recent systematic review was shown to be associated with poorer cognition in aging (6), likely due to accompanying neuroinflammation. Therefore, dietary patterns that maintain healthy gut function during aging, may limit this inflammatory cascade and reduce the risk of the development of neurodegeneration.

Several studies have attempted to correlate dietary patterns with cognition in later life, and many of these have identified dairy as being part of a cognitively healthy diet. The majority of these studies were conducted in developed Asian countries that have a relatively high prevalence of neurodegenerative diseases (7) and relatively low intake of dairy products (8), for example, Japan, South Korea (9, 10), Taiwan (11), Singapore (12) and China (13, 14). However, in these dietary pattern studies is it difficult to determine the contribution of dairy consumption to any cognitive outcomes.

This review evaluated the literature to determine whether there is sufficient evidence to suggest that the consumption of dairy products at any life stage helps to maintain cognitive function in later life. The process used to identify relevant studies and the findings are discussed below. A search was conducted on 28/6/2022 using the Web of Science, PubMed, PsychINFO, and Scopus databases. The search terms used were: (dairy OR milk OR cheese OR yogurt OR yogurt) AND (“mild cognitive impairment” OR dementia OR “Alzheimer's disease”). After the removal of duplicates, 796 articles were identified. The articles from the search were screened for relevance, primarily based on their titles. If the content of the article was not clear from the title, then the abstract was reviewed. Based on this, 574 articles were excluded because they were not directly relevant to the topic. Articles were excluded if they focused on other age groups (e.g., infant/child cognitive development, early-onset dementia), other diseases (e.g., Parkinson's disease, multiple sclerosis), or other food products (e.g., milk thistle, probiotics). Articles were also excluded if the main text was not in English or if they were abstracts for conferences without an associated full-text article.

The abstracts of the remaining 222 studies were read and classified into research summaries (literature reviews and meta-analyses) and original research. The original research was separated into pre-clinical studies (animal studies and *in vitro* studies) and human studies. A further 37 articles were excluded as irrelevant because they focused on other topics (e.g., chemical analyses of dairy products). The remaining 51 human studies identified were evaluated in detail. These were categorized as observational studies that aimed to correlate dairy intake with cognition in older adults, and experimental studies that aimed to improve cognition in older adults with dairy interventions. Another 22 studies were excluded because they focused on dietary patterns where it was difficult to isolate the impact of dairy, or they had outcome measures that did not include a cognitive test (e.g., brain imaging). The final 23 observational studies and six intervention studies identified are evaluated in the following sections.

## Observational studies

There have been numerous attempts to find links between dairy consumption and cognition during aging using observational studies. In the 23 studies identified by this literature search, three study designs were used. The least convincing are cross-sectional studies where dietary intake and cognition are measured at the same time. Without temporal precedence, it is not possible to determine whether any identified associations mean that the diet affected cognition or whether changes in cognition altered dietary choices. In addition, participants experiencing cognitive impairment may not be able to accurately recall their dietary intake. An improvement on this is retrospective cohort studies where data about previous dietary intake is collected from records and compared to current cognition. However, these have limitations such as the data may be of poor quality because it was not collected for this purpose. Finally, the most robust observational studies are prospective cohort studies where dietary intake is assessed at one point in time then cognitive function is assessed later.

In many of the published observational studies, dairy products were pooled together as one category with no distinction between the types of products. However, some studies focused on milk alone, high-fat dairy or fermented dairy. The observational studies related to these four categories are discussed in the subsections below. Some studies are discussed in multiple subsections because they contain data from different product categories.

### Total dairy products

The observational studies that assessed total dairy product intake collectively are summarized in Table 1 in the order in which they were published. Ten were cross-sectional studies and four were prospective cohort studies.

**Table 1:** Summary of observational studies assessing total dairy intake and cognitive function.

**Participants**	**Numbers**	**Design**	**Diet measures**	**Cognitive tests**	**Outcome**	**Result**	**References**
60+ years; living in South Korea	449 (210 men, 239 women)	Cross-sectional study; single in-person session with each participant	24-h dietary recall, food habits questionnaire	MMSE-Koreans	Positive/no association	Women with MMSE ≥ 24 had higher dairy intake than those with MMSE ≤ 19 (Cohen's *d* = 0.56^a^) No significant difference in dairy intake between men with different MMSE scores (*P* > 0.05)	Lee et al. (15)
65+ years; living in Taiwan	1,839 (925 men, 914 women)	Cross-sectional study; single in-person session with each participant	24-h dietary recall, Diet Diversity Score	Short Portable Mental Status Questionnaire	Positive	More people with intact cognition consumed dairy daily than those with impaired cognition (43 vs. 37%, *P* < 0.05)	Chen et al. (16)
60+ years; living in the US	6,471	Cross-sectional study; single in-person session with each participant (from survey records)	24-h dietary recall	Story recall test (4,282 participants), digit-symbol substitution test (2,189 participants)	Positive	Those who consumed dairy had higher story recall scores than those who did not (Cohen's *d* = 0.16^a^)	Park and Fulgoni (17)
60+ years at study start; living in Japan	570 (298 men, 272 women)	Prospective study; diet was assessed then cognition was measured every 2 years for 10 years	3-day dietary record	MMSE-Japanese	Positive/no association	A 1 SD increase in dairy consumption reduced odds of MCI in women (AOR = 0.77, *P* = 0.007) No significant effect of dairy intake in men (*P* = 0.516 for AOR)	Otsuka et al. (18)
60+ years at study start; living in Japan	1,081 (457 men, 624 women)	Prospective study; diet was assessed then cognition was measured every 2 years for 10 years	70-item semi-quantitative FFQ	MMSE-Japanese, Hasegawa Dementia Scale, Hasegawa Dementia Scale-Revised	Positive	Likelihood of developing dementia was reduced in those who consumed moderate dairy (Q1 compared to Q3 intake HR = 0.66, CI = 0.48–0.91, *P* < 0.03)	Ozawa et al. (19)
65+ years; living in Central Africa	1,772 (725 men, 1,047 women)	Cross-sectional study; single in-person session with each participant	8-item FFQ	Community Screening Interview for Dementia; Free and Cued Selective Reminding Test, Zazzo's cancellation task, Isaac's Set Test of verbal fluency	No association	No association between dairy consumption and dementia (*P* = 0.133 for AOR)	Pilleron et al. (20)
50+ years; living in South Korea	276 (105 men and 171 women)	Cross-sectional study; single in-person session with each participant	112-item FFQ	MMSE	Positive	Increased daily dairy consumption reduced odds of MCI (AOR = 0.100, *P* = 0.003)	Kim and Yun (21)
60+ years; living in the Netherlands	619 (369 men, 350 women)	Cross-sectional study; single in-person session with each participant (baseline data from intervention study)	190-item FFQ	MMSE, Digit Span forward and backward, Trail Making Test, troop Color-Word Test, Letter Fluency, Symbol Digit Modalities Test, Rey Auditory Verbal Learning Test	No association	No association between total dairy consumption and any of the cognitive domains tested (*P* > 0.05 for β and OR)	de Goeij et al. (22)
60+ years; living in the US	2,660 (1,167 men, 1,293 women)	Cross-sectional study; single in-person session with each participant	Two 24-h diet recall interviews	Word List Learning Test and Word List Recall Test from the Consortium to Establish a Registry for Alzheimer's disease, Animal Fluency Test, Digit Symbol Substitution Test	Negative	High dairy intake associated with lower composite scores (Q1 vs. Q2–4 weighted regression co-efficient (β) = −0.13, 95%CI: −0.24 to −0.02)	Li et al. (23)
60+ years at end of study; living in Singapore	17,107 (7,073 men, 10,034 women)	Prospective study; diet was assessed then cognition was measured every three times over 20 years	165-item FFQ, 24-h diet recall interview	Singapore Modified MMSE	Positive	Higher dairy consumption reduced odds of MCI (Q3 and Q4 vs. Q1 AOR = 0.84 and 0.79, *P* = 0.004)	Talaei et al. (24)
55+ years; living in China	4,309 (1,956 men, 2,353 women)	Cross-sectional study; single in-person session with each participant (baseline data for longitudinal study)	81-item FFQ	Montreal Cognitive Assessment	Positive	Moderate dairy consumption reduced odds of MCI (Q1 vs. Q3 AOR = 0.65, *P* < 0.05)	Huang et al. (25)
55+ years; living in Spain	6,426 (3,158 men, 3,288 women)	Cross-sectional study; single in-person session with each participant (baseline data for longitudinal study)	146-item FFQ	MMSE	Negative	High dairy consumption increased odds of MMSE ≤ 26 (Q1 vs. Q4 AOR = 1.40, *P* = 0.001)	Muñoz-Garach et al. (26)
65+ years; living in Canada	7,945 (4,079 men, 3,866 women)	Cross-sectional study; single in-person session with each participant (baseline data for longitudinal study)	36-item short diet questionnaire	15-word Rey Auditory Verbal Learning Test immediate and delayed recall, Mental Alternation Test, high interference of the Victoria Stroop test (interference/dot), event- and time-based prospective memory tests, and 2 verbal fluency tests, Animal Fluency Test and Controlled Oral Word Association Test of the letters, choice reaction time	Positive	Higher dairy intake associated with higher executive function scores (partial η^2^ = 0.001, *P* = 0.012)	Tessier et al. (27)
42+ years at start of study; living in Finland	1,741 men	Prospective study; diet was assessed and then cognition was measured 4 years later	4-day food recording	Dementia diagnosis (1,259 participants); MMSE, trail making test A, verbal fluency test, selective reminding test, Russell's adaptation of the visual reproduction test (482 participants 60+ years)	No association	Total dairy intake not associated with dementia risk (*P* = 0.21 for AOR) or cognitive test scores (*P* > 0.05 for comparisons between intake groups)	Ylilauri et al. (28)

Green indicates a positive association between total dairy intake and cognition, red indicates a negative association and black indicates no association.

AOR, Adjusted odds ratio; FFQ, Food frequency questionnaire; HR, Hazard ratio; MMSE, Mini-Mental State Exam; Q1-Q4, Quintile 1–4 intake groups.

^a^Cohen's d calculated based on data given in the publication.

#### Cross-sectional studies

Of the 10 cross-sectional studies that correlated participants' current diet with cognition outcomes, six showed a positive association between dairy intake and cognition. Two of these studies were conducted in South Korea. In the first study, the prevalence of low cognition (MMSE ≤ 19) was greater in women than in men (31% vs. 22%) (15). The intake of dairy products by women in the low cognition group was significantly lower than those in the normal cognition group (MMSE ≥ 24) (Cohen's *d* = 0.56). In the men, there was no significant difference in dairy product consumption between the low vs. normal cognition groups. In the second study, cognitively healthy individuals were compared to those with MCI (MMSE = 20–23) (21). Those who had adequate dairy consumption were 90% less likely to have MCI [adjusted odds ratio (AOR) = 0.100].

Studies in other Asian countries yielded similarly positive results. For example, in a study conducted in Taiwan, a higher percentage of those with intact cognition had a dairy food intake score of one per day compared to those with impaired cognition (43% vs. 37%) (16). Consistent with that, a Chinese study found that higher dairy intake reduced the odds of MCI by 35% overall (25). However, they found that the effect of dairy intake had mixed effects for different subtypes of MCI, with a higher dairy intake being associated with increased odds of amnestic MCI (OR = 1.51) but reduced odds of non-amnestic MCI (OR = 0.49).

An additional two studies found a positive association between dairy intake and cognition; however, the effect sizes were smaller than those observed in the studies with Asian populations. In a study based in the US, those who consumed dairy products had slightly better episodic memory than those who did not (Cohen's *d* = 0.16) (17). In addition, a Canadian study found a statistically significant association between total dairy intake and executive function score, however, the effect size was very small (partial η^2^ = 0.001) (27).

Two of the 10 cross-sectional studies did not show any correlation between dairy intake and cognition. This included a study conducted in Central Africa where there was no difference in the incidence of dementia between those who consumed dairy products at least once a day and those who did not (20). This study was quite simplistic in its data analysis—absence or presence of dementia and regular or not dairy consumption. It is possible that a more nuanced approach, with levels of cognition and dairy consumption, is required to detect a relationship. However, a more complex Dutch study, which looked at the correlation between dairy intake and scores in several cognitive domains, also found no relationships (22).

Finally, two of the cross-sectional studies found negative associations between total dairy intake and cognition. A study conducted in the US found that those who consumed high amounts of dairy products (Q4) had lower composite scores compared to those who consumed fewer dairy products (Q1) when results were controlled for potential confounding factors (β = −0.13) (23). Although the effect size of the composite score is small, those of the recall and delayed recall tests were medium (β = −0.27) and large (β = −0.6), respectively. Similarly, in a study completed in Spain, high dairy intake (Q4) was associated with a 40% increase in the odds of cognitive decline (26).

The discrepancies between the results of the studies appear to be due to differences in the dairy intakes between the different study populations. For the studies where positive effects of dairy were observed the average intakes tended to be lower than those where a negative effect was observed. The studies conducted in Asian countries, which had the largest effect sizes, reported the lowest intakes: averages of 35 g/day (15), 0.9 times/day (21), and 77 g/day (25) [daily dairy intake was not provided by Chen et al. (16)]. Whereas, in the studies that reported very small positive effects the intakes were higher: median 78 g/day and Q4 > 163 g/day (17), and median 1.9 times per day and Q4 > 2.5 times/day (27). In comparison, in the two studies in which dairy consumption had a negative correlation, the average intakes were much higher. The analysis by Li et al. (23) focused on protein intake and compared the Q4 > 12.8 g protein/day (equivalent to 376 ml milk/day) group to the Q1 < 4 g protein/day (equivalent to 117 ml milk/day). Whereas, in the study conducted by 24, the Q1 and Q4 intakes were <200 g/day and ≥500 g/day (26). The total dairy intake of the groups that had higher cognition compared to those that had lower cognition are summarized in Table 2. Collectively, these results indicate that the relationship between dairy intake and cognition is an inverse U-shape, where a moderate amount of dairy consumption of 1–2 servings of dairy per day is optimum for cognitive function.

**Table 2:** Summary of total dairy intake of those who had higher cognitive outcomes (optimum) compared to the intakes of those who had lower cognitive outcomes (too low or too high).

**Study**	**Intake too low**	**Optimum intake**	**Intake too high**
Chen et al. (16)	<once/day	≥once/day	
Park and Fulgoni (17)	No dairy	Some dairy	
Ozawa et al. (19)	Q1 and Q2 = 0–96 g/day	Q3 = 97–197 g/day	Q4 = 198+ g/day
Pilleron et al. (20)	<once/day	≥once/day	
Talaei et al. (24)	Q1, Q2, Q3 medians = 6, 9 and 37 g/day	Q4 median = 252 g/day	
Huang et al. (25)	Q1 and Q2 = 0–100 g/day	Q3 = 150–200 g/day	Q4 = 300–450 g/day
Muñoz-Garach et al. (26)		Q1-Q3 = 0–500 g/day	Q4 > 500 g/day
Tessier et al. (27)	Q1 and Q2 < 1.7 times/day	Q3 and Q4 ≥ 1.7 times/day	

#### Prospective cohort studies

The other published observational studies assessing links between dairy product intake and cognition in later life were prospective cohort studies. Three of these found that dairy consumption reduced the risk of cognitive impairment. For example, in a study based in Japan, women who consumed more dairy products had, 23% reduced odds of developing cognitive decline over the next 10 years than those who consumed fewer dairy products (18). This association was not observed in men.

In another Japanese study, the benefits of dairy products were dependent on the intake level (19). Compared to those who had a low dairy intake (Q1: <45 g/day for women and <20 g/day for men), those who had a moderate intake (Q3: 97–197 g/day for women and 76–173 g/day for men) had a reduced overall likelihood of developing dementia during the 17-year study period [adjusted hazard ratio (HR) = 0.66]. However, the effects were not significant for the Q2 or Q4 intake groups, which supports the idea that the relationship between dairy intake and cognitive benefit is an inverse U-shape.

Those studies have been followed up more recently with a study in Singapore involving a much larger cohort (24). The dairy consumption in the study population was very low, with the median daily intakes of Q1-Q4 being 5.68, 9.41, 36.6, and 252 g/day. The Q3 and Q4 intake groups had decreased odds of cognitive impairment of 16 and 21%, respectively, over the 20-year study period.

In contrast, one study did not find any association between total dairy intake and the risk of developing dementia or performance in cognitive tests (28). This study was conducted in Finland, so again different results were observed in a Western country compared to Asian countries. This is likely due to the dairy intake which was much higher than that in the previously discussed studies: Q1 was <455 g/day and Q4 was >927 g/day. The median intake in the Q1 group (292 g/day) was higher than the median intake in the Q4 group in the study by Talaei et al. (24).

Although the direction of associations between dairy intake and later-life cognition appears to be dependent on the intake range in the study population, another explanation for the discrepancies between studies could be due to the types of dairy products consumed in the test populations. Therefore, in the following subsections associations between different types of dairy products and cognition are explored.

### Milk

Ten of the observational studies examined milk consumption. In all cases, the species of milk was not described, but it is assumed that the majority is bovine milk, which contains 4.6% lactose, 3.4% protein, and 4.2% fat, as well as 0.8% minerals and 0.1% vitamins. The experimental details of these studies are summarized in Table 3. Five studies used a cross-sectional study design, one was a retrospective cohort study and four were prospective cohort studies.

**Table 3:** Summary of observational studies assessing milk intake and cognitive function.

**Participants**	**Numbers**	**Design**	**Diet measures**	**Cognitive tests**	**Outcome**	**Results**	**References**
60+ years at end of study; living in Japan	1,774 (475 men, 1,299 women)	Prospective study; diet was assessed during mid-life and then cognition was measured 25–30 years later	Food consumption frequencies	CASI for all; Informative Questionnaire on Cognitive Decline in the Elderly, Hachinski's Ischemic Score and Clinical Dementia Rating for a subset of participants	Positive	Daily milk intake reduced likelihood of vascular dementia compared to milk consumption less than twice a week (OR = 0.26, *P* = 0.002)	Yamada et al. (29)
80+ years at end of study; living in Australia	857 men	Prospective study; diet was assessed and then cognition was measured 5 years later	Diet questionnaire	MMSE	Negative	Regular consumption of milk reduced likelihood of MMSE score ≥25 (OR = 0.69, *P* < 0.05)	Almeida et al. (30)
Elderly (age not defined); from the US	1,056 (345 men, 711 women)	Cross-sectional study; single in-person session with each participant	10-item FFQ	Mental status questionnaire		No association between milk intake and cognitive scores (*P* = 0.18 for AOR)	Rahman et al. (31)
50–82 at end of study; living in China	2,062 (1,051 men, 1,011 women)	Retrospective study	Childhood data extracted from medical records	Battery including Flud object memory evaluation, Fuld verbal fluency, WISC-R, WAIS-R digit span	Positive	Daily milk intake during childhood reduced risk of low combined cognitive score in later life (AOR = 0.64, *P* < 0.001)	Zhang et al. (32)
60+ years; living in the US	6,471	Cross-sectional study; single in-person session with each participant (from survey records)	24 h dietary recall	Story recall test (4,282), digit-symbol substitution test (2,189)	No association	No differences in cognitive test scores between milk intake groups (*P* > 0.05)	Park and Fulgoni (17)
70+ years in at end of study; living in the US	5,987	Prospective study; diet was assessed twice and cognition was measured three times over 25 years	FFQ	Decall Word Recall Test, Digit Symbol Substitution Test, Word Fluency Test	Negative	Daily milk intake associated with lower global cognition scores compared to almost never consumption (*z* score = −1.04 vs. −0.94)	Petruski-Ivleva et al. (33)
50+ years; living in South Korea	276 (105 men, 171 women)	Cross-sectional study; single in-person session with each participant	112-item FFQ	MMSE	Positive	Increased milk consumption reduced odds of MCI (AOR = 0.421, *P* = 0.044)	Kim and Yun (21)
60+ years; living in the Netherlands	619 (369 men, 350 women)	Cross-sectional study; single in-person session with each participant (baseline data for longitudinal study)	190-item FFQ	MMSE, Digit Span forward and backward, Trail Making Test, Stroop Color-Word Test, Letter Fluency, Symbol Digit Modalities Test, Rey Auditory Verbal Learning Test	No association	No association between total milk consumption and any of the cognitive domains tested (*P* > 0.05 for β and AOR)	de Goeij et al. (22)
65+ years; living in Canada	7,945 (4,079 men, 3,866 women)	Cross-sectional study; single in-person session with each participant (baseline data for longitudinal study)	36-item short diet questionnaire	15-word Rey Auditory Verbal Learning Test immediate and delayed recall, Mental Alternation Test, high interference of the Victoria Stroop test (interference/dot), event- and time-based prospective memory tests, and 2 verbal fluency tests, Animal Fluency Test and Controlled Oral Word Association Test of the letters, choice reaction time	No association	No association between milk intake and cognitive test scores (*P* > 0.05 for partial η^2^)	Tessier et al. (27)
42+ years at start of study; living in Finland	1,741 men	Prospective study; diet was assessed and then cognition was measured 4 years later	4-day food recording	Dementia diagnosis (1,259 participants); MMSE, trail making test A, verbal fluency test, selective reminding test, Russell's adaptation of the visual reproduction test (482 participants 60+ years)	Negative/No association	No association between milk intake and dementia or AD risk (*P* = 0.18 and 0.20) High milk intake group had lower verbal fluency test scores than low milk intake group (30.6 vs. 33.5, *P* = 0.03)	Ylilauri et al. (28)

AOR, Adjusted odds ratio; CASI, Cognitive Ability Screening Instrument (contains items similar to MMSE and Hasegawa's Dementia Scale); FFQ, Food frequency questionnaire; MMSE, Mini-Mental State Exam; WISC-R, Wechsler Intelligence Scale for Children-Revised; WAIS-R, Wechsler Adult Intelligence Scale-Revised.

#### Cross-sectional studies

Four of the cross-sectional studies did not find any association between milk intake and cognition. Park and Fulgoni (17) did not find differences in scores for the cognitive domains tested between those who had no, low, moderate or high milk consumption. Similarly, Rahman et al. (31), Tessier et al. (27), and de Goeij et al. (22) did not find any change in the risk of low cognitive test scores associated with milk intake. In two of the studies total dairy consumption was associated with higher cognitive test scores (17, 27), so this implies that the observed benefits were due to dairy products other than milk.

In contrast, one of the cross-sectional studies found a relationship between milk intake and cognition. Kim and Yun (21) observed that increasing the once-per-day frequency of milk consumption reduced the risk of MCI by 58%. However, the average milk intake in this study was only 0.36 servings/day. In comparison, in the Tessier et al. (27) study the average milk intake was 0.89 servings/day. The other two studies that did not find associations between milk intake and cognition did not give data on the daily milk intake ranges (17, 22). Nevertheless, the total dairy intakes were higher in those studies conducted in Western populations than in the South Korean study (21). Therefore, the results suggest that an increase from low to adequate milk consumption is beneficial, but no additional benefits occur with higher than adequate milk intake.

#### Retrospective cohort studies

Unlike the previous studies presented that assessed current dietary intake, a retrospective cohort study was conducted in China where childhood medical data was collated to look for relationships between childhood diet and cognition in later life (32). The results showed that after adjusting for various potential influencing factors, daily milk consumption during childhood reduced the likelihood of lower cognition later in life by 36%. Unfortunately, there are no other published studies focusing on the role of milk consumption in childhood in later-life cognition to confirm this result.

#### Prospective cohort studies

Four prospective cohort studies recorded milk intake during mid-life, followed by cognitive assessment in later life. The results of these are conflicting. In a Japanese study, those who consumed milk almost daily during mid-life had a 74% lower likelihood of developing vascular dementia in later life than those who consumed milk less than twice a week (29). There was no significant association between milk consumption and AD development.

In contrast, in the other three prospective studies, increased milk consumption during mid-life was linked to worse cognitive outcomes in later life. In an Australian study with men only, regular consumption of milk increased the likelihood of cognitive impairment (MMSE < 24) 5 years later by 31% (30). Similarly, a study conducted in the US determined that those who consumed milk daily had on average 11% greater cognitive decline over 20 years than those who rarely consumed milk (33). Finally, the Finnish study previously described found no change in dementia or AD risk associated with milk intake; however, they found that the average verbal fluency test scores were slightly lower (8.6%) in those in the high milk intake group compared to the low intake group (28).

Unlike comparisons between studies previously discussed, there is no difference in the intake levels that may account for the differences in the results. Two of the studies that found negative associations between mid-life milk intake and later-life cognition compared regular/daily milk consumption with rarely/no milk consumption (30, 33), the same as the comparison made by Yamada et al. (29) who found higher milk consumption had a protective effect.

There are several differences in the prospective cohort study designs that may account for the differences in the results. For example, the measures used to assess cognition and the definitions of impaired cognition were different between the studies, so the results are not directly comparable. Ylilauri et al. (28) and Yamada et al. (29) primarily examined the relationship between diet and clinically diagnosed dementia; whereas, Almeida et al. (30) included those with mild cognitive impairment (MMSE < 24) and Petruski-Ivleva et al. (33) looked at changes in cognition over time. This implies that milk may have a protective effect against the development of vascular dementia or AD, but not for MCI, although more research is required to confirm this.

Another factor that may have influenced the results is gender. The studies by Ylilauri et al. (28) and Almeida et al. (30) included men only, and the study by Petruski-Ivleva et al. (33) did not give details on the genders of the participants; whereas, the study by Yamada et al. (29) 67% of the participants were women. This may indicate that higher milk consumption is more beneficial for maintain cognition in women than men, which is worth further enquiry.

### Dairy lipids

One factor that may account for the inconsistencies in the results between observational studies in different populations may be whether full-fat or skim dairy products are more commonly consumed. In the human brain, ~50% of the dry weight is lipids, and the ingestion of dairy lipids during early life is known to be critical for infant brain development (34). Therefore, it is plausible that dairy lipid consumption could also be important for maintaining cognition during aging. Seven studies were identified in the literature review to have investigated the role of dairy lipids in later-life cognition. These studies are summarized in Table 4 and discussed below in subsections separating studies that compared full-fat to skim dairy intake, high-fat dairy desserts, and those that examined lipid compositions.

**Table 4:** Summary of observational studies assessing dairy lipid intake and cognitive function.

**Participants**	**Numbers**	**Design**	**Diet measures**	**Cognitive tests**	**Outcome**	**Results**	**Reference**
65–79 years at end of study; from Finland	1,341 (596 men, 835 women)	Prospective study; diet was assessed and then cognition was measured 10–25 years later	20-item FFQ	MMSE, dementia/AD diagnosis	Positive/negative	Likelihood of developing dementia was reduced in those who consumed moderate dairy lipids (Q2 vs. Q1 AOR = 0.43, *P* < 0.05) Moderate intake of dairy PUFA and MUFA had protective effects (Q2 and Q3 vs. Q1 AOR = 0.48 and 0.51 for dementia; Q3 vs. Q1 AOR = 0.36 for AD, *P* < 0.05) Moderate intake of dairy SFA increased risk of dementia and AD (Q2 vs. Q1 AOR = 1.76 for dementia; Q3 vs. Q1 AOR = 2.29 for AD, *P* < 0.05)	Laitinen et al. (35)
65–79 years at end of study; from Finland	1,341 (596 men, 835 women)	Prospective study; diet was assessed and then cognition was measured 10–25 years later	20-item FFQ	MMSE, word recall tests, The Category Fluency Test, Purdue Peg Board task, letter digit substitute test, Stroop test	Negative	High dairy lipid and SFA consumption in mid-life associated with MCI (AOR = 1.69 and 2.36 adjusted for MCI risk factors) High dairy lipid intake group had lower MMSE and psychomotor speed scores (Cohen's *d* = 0.12 and 0.33^a^) High SFA intake group had lower MMSE and prospective memory scores (Cohen's *d* = 0.15 and 0.05^a^)	Eskelinen et al. (36)
76–82 years at end of study; from France	4,809 women	Prospective study; diet was assessed and then cognition was measured 13 years later	208-item diet history questionnaire	“DEte'rioration Cognitive Observe'e” (DECO, observed cognitive deterioration) score; IADL	Negative	High intake of dairy desserts and ice cream associated with higher risk of cognitive decline compared to no intake (AOR = 1.33 for observed cognitive deterioration score)	Vercambre et al. (37)
60+ years; living in Brazil	400 (112 men, 288 women)	Cross-sectional study; single in-person session with each participant	Weekly consumption estimates	MMSE	No association	No difference in risk of cognitive deficit in those who consumed whole vs. skim milk (*P* = 0.998 for AOR)	Franca et al. (38)
60+ years; living in the Netherlands	619 (369 men, 350 women)	Cross-sectional study; single in-person session with each participant (baseline data from intervention study)	190-item FFQ	MMSE, Digit Span forward and backward, Trail Making Test, troop Color-Word Test, Letter Fluency, Symbol Digit Modalities Test, Rey Auditory Verbal Learning Test	No association	Association between skim dairy intake and executive function (β = 0.07, *P* = 0.01), but not full-fat dairy (*P* > 0.05) No association between full-fat or semi-skim/skim milk consumption and any of the cognitive domains tested (*P* > 0.05 for β)	de Goeij et al. (22)
55+ years; living in Spain	6,426 (3,158, 3,288 women)	Cross-sectional study; single in-person session with each participant (baseline data for longitudinal study)	146 item FFQ	MMSE	Positive	Moderate full-fat milk and dairy consumption decreased odds of MMSE ≤ 26 (Q2 vs. Q1 AOR = 0.73, *P* = 0.001; Q3 vs. Q1 AOR = 10.74, *P* = 0.003)	Muñoz-Garach et al. (26)
65+ years; living in Canada	7,945 (4,079 men, 3,866 women)	Cross-sectional study; single in-person session with each participant (baseline data for longitudinal study)	36-item short diet questionnaire	15-word Rey Auditory Verbal Learning Test immediate and delayed recall, Mental Alternation Test, high interference of the Victoria Stroop test (interference/dot), event- and time-based prospective memory tests, and 2 verbal fluency tests, Animal Fluency Test and Controlled Oral Word Association Test of the letters, choice reaction time	No association	No association between full-fat dairy intake and cognitive test scores in adjusted models (*P* > 0.05 for all partial η^2^)	Tessier et al. (27)

AOR, Adjusted odds ratio; FFQ, Food frequency questionnaire; HR, Hazard ratio; IADL, Instrumental activities of daily living; MMSE, Mini-Mental State Exam; MUFA, Monounsaturated fatty acids; PUFA, Polyunsaturated fatty acids; SFA, Saturated fatty acids; Q1-Q4, Quintile 1–4 intake groups.

^a^Cohen's d calculated based on data given in the publication.

#### Full-fat dairy

Four cross-sectional studies investigated the associations between full-fat and skim dairy products separately on cognition and the results were mixed. In the study conducted by Muñoz-Garach et al. (26), which found higher total dairy intake was associated with an increased risk of MCI, moderate full-fat milk and dairy product intake reduced the likelihood of cognitive impairment (27% for Q2 and 26% for Q3 compared to Q1). This implied that the lipids may be responsible for the protective effects.

In contrast, the results from two other studies indicate that components other than lipids were responsible for any cognitive benefits of dairy consumption. In the de Goeij et al. (22) study, which found no correlations between total dairy or milk intake and cognitive scores, a small correlation between skim dairy intake and executive function was observed (β = 0.07), but not full-fat dairy. Similarly, Tessier et al. (27) found higher low-fat dairy intake was associated with slightly higher executive function scores (partial η^2^ = 0.002), and there was no difference in scores between full-fat dairy intake groups.

The results of the three studies described above were potentially confounded by the inclusion of fermented dairy products. When comparing total milk, semi-skim/skim and full-fat milk intakes where the only difference between products was the lipid content, de Goeij et al. (22) found no associations with any cognitive domain scores. In support of this, there were no differences in cognitive function between those consuming skim vs. full-fat milk in a study based in Brazil (38).

#### High-fat dairy desserts

In a French study that included only women, the researchers analyzed high-fat dairy desserts separate from other dairy products; however, the results were inconclusive due to confounding factors. Those who had high intakes of dairy desserts and ice cream in their 60s (>median consumption) were 33% more likely to develop cognitive impairment in later life compared to those who did not consume these products (37). The association with the intake of pastries and cakes was almost significant (*P* = 0.056). Dairy desserts and ice cream, as well as pastries and cakes, are high in refined sugars; therefore, it is not possible to assign the association solely to dairy lipids. This is in agreement with the study by Tessier et al. (27) where the authors excluded dairy desserts from the analysis of total dairy products due to the high refined sugar content potentially impacting the results.

#### Lipid composition

Despite the necessity of lipids for brain structure and function, a research group from Finland hypothesized that high fat and cholesterol intake from dairy consumption in mid-life was a risk factor for the development of dementia and AD in later life (35). However, analysis of the impact of total dairy lipid intake did not support the hypothesis. Those with moderate intake of lipids from dairy products and spreads (Q2: 18.7–30.2 g/day) were less likely to develop dementia compared to those with a low intake (Q1: <18.6; AOR = 0.43). There were no changes in the likelihood of dementia in the Q3 and Q4 groups compared to Q1, again indicating that moderate intake is optimum. In addition, there was no correlation between total dairy lipid intake in mid-life and AD in later life.

However, further analysis showed the type of lipids consumed was important to the outcomes. For dementia, compared to low intake (Q1), moderate intake of polyunsaturated fatty acids (PUFA) and mono-unsaturated fatty acids (MUFA) had protective effects (AOR = 0.48 and 0.51 for Q2); whereas moderate intake of saturated fatty acids (SFA) was a risk factor (AOR = 1.76 for Q2). Similarly, for AD, moderate intake of PUFA had protective effects (AOR = 0.36 for Q3) and SFA intake was a risk factor (AOR = 2.29 for Q2). The risk associated with SFA consumption was increased if the model was adjusted for other cognitive decline risk factors including the AD-associated genotype (ApoE e4; AOR = 2.45 for Q2 for dementia; AOR = 3.82 for Q2 for AD). Surprisingly, a higher intake of SFA (Q3 and Q4) did not alter the likelihood of developing either dementia or AD compared to a low intake (Q1). The reason for this is unclear.

Data from the same study were analyzed in a separate publication focusing on MCI in later life (36). In that analysis, increased intake of milk lipids, PUFA and MUFA did not alter the likelihood of the development of MCI. However, the analysis compared high (Q3 and Q4) and low (Q1 and Q2) intake only, so the benefits of moderate consumption (Q2 vs. Q1) observed in the previous analysis were not assessed. There was an increased likelihood of later-life MCI based on higher SFA consumption in mid-life (AOR = 2.25), and when adjusted for MCI risk factors there were significant associations for both total lipid and SFA consumption (AOR = 1.69 and 2.36). In the adjusted model, those with higher total lipid intake had lower MMSE and psychomotor speed scores although the effect sizes were small (Cohen's *d* = 0.12 and 0.33). In addition, those with higher SFA intake had slightly lower MMSE and prospective memory scores (Cohen's *d* = 0.15 and 0.05).

Collectively, the results from both publications provide some support for the hypothesis that high dairy SFA intake is a risk factor for the development of MCI, dementia and AD, although the effect sizes were negligible. As this study focused primarily on dairy intake, it is unclear how the results may have been influenced by the intake of other lipids in the diet, particularly SFA from other animal sources (meat and eggs). It is also important to note that some lipids found in milk (PUFA and MUFA) were associated with cognitive benefits. Further research is required to understand the optimum dairy lipid consumption profile to maximize any beneficial effects.

### Fermented dairy products

Fermented dairy products are made by the microbial fermentation of dairy products. The most common are yogurt and cheese, however, it also includes products such as buttermilk, sour cream, and crème fraiche. In most cases, the fermentation is carried out by lactic acid bacteria, many of which are considered beneficial to health. Therefore, it is difficult to distinguish whether any effects of fermented dairy products are due to the dairy, remaining live microbes, or inactivated microbial factions such as cell surface molecules. Seven studies that investigated the role of fermented dairy products on cognitive function in later life are summarized in Table 5. They are separated into subsections below based on the type of fermented dairy product.

**Table 5:** Summary of observational studies assessing fermented dairy product consumption and cognitive function.

**Participants**	**Numbers**	**Design**	**Diet measures**	**Cognitive tests**	**Outcome**	**Results**	**References**
Elderly (age not defined); from the US	1,056 (345 men, 711 women)	Cross-sectional study; single in-person session with each participant	10-item FFQ	Mental status questionnaire	Positive	Cheese consumption at least weekly reduced likelihood of mental status score of < 9 (AOR = 0.68, *P* = 0.47)	Rahman et al. (31)
60+ years; living in the US	6,471	Cross-sectional study; from survey records of single in-person session with each participant	24 h dietary recall	Story recall test (4,282), digit-symbol substitution test (2,189)	Positive	Those who consumed cheese had higher story recall test scores (Cohen's *d* = 0.12^a^)	Park and Fulgoni (17)
50+ years; living in South Korea	276 (105 men, 171 women)	Cross-sectional study; single in-person session with each participant	112-item FFQ	MMSE	Positive	Increased liquid and curd yogurt consumption reduced odds of MCI (AOR = 0.264, *P* = 0.019 for liquid and AOR = 0.263, *P* = 0.015)	Kim and Yun (21)
60+ years; living in the Netherlands	619 (369 men, 350 women)	Cross-sectional study; single in-person session with each participant (baseline data from an intervention study)	190-item FFQ	MMSE, Digit Span forward and backward, Trail Making Test, troop Color-Word Test, Letter Fluency, Symbol Digit Modalities Test, Rey Auditory Verbal Learning Test	Positive/no association	Fermented dairy consumption associated with increased executive function (β = 0.06, *P* = 0.03) Buttermilk consumption associated with increased executive function (β = 0.1, *P* = 0.03) Dutch cheese consumption reduced risk of low cognitive processing speed (HR = 0.67) No association between total yogurt or cheese consumption and any cognitive domain (*P* > 0.05 for β)	de Goeij et al. (22)
46–77 years old at end of study; living in the UK	3,113 (1,634 men, 1,479 women)	Prospective study; diet and cognition were assessed at baseline, 5 years and 10 years	FFQ	Fluid Intelligence Test	Positive	Higher cheese intake associated with better test scores (β = 0.207, *P* < 0.0001)	Klinedinst et al. (39)
55+ years; living in Spain	6,426 (3,158, 3,288 women)	Cross-sectional study; single in-person session with each participant (baseline data from an intervention study)	146 item FFQ	MMSE	Negative	High fermented dairy consumption increased odds of MMSE ≤ 26 (Q4 vs. Q1 AOR = 1.34, *P* = 0.003)	Muñoz-Garach et al. (26)
65+ years; living in Canada	7,945 (4,079 men, 3,866 women)	Cross-sectional study; single in-person session with each participant (baseline data from an intervention study)	36-item short diet questionnaire	15-word Rey Auditory Verbal Learning Test immediate and delayed recall, Mental Alternation Test, high interference of the Victoria Stroop test (interference/dot), event- and time-based prospective memory tests, and 2 verbal fluency tests, Animal Fluency Test and Controlled Oral Word Association Test of the letters, choice reaction time	Positive	Higher fermented dairy and cheese intakes associated with higher executive function scores (partial η^2^ = 0.002 and 0.001, *P* < 0.001) Higher yogurt intake associated with higher memory scores (partial η^2^ = 0.001, *P* < 0.05)	Tessier et al. (27)
42+ years at start of study; living in Finland	1,741 men	Prospective study; diet was assessed and then cognition was measured 4 years later	4-day food recording	Dementia diagnosis (1,259 participants); MMSE, trail making test A, verbal fluency test, selective reminding test, Russell's adaptation of the visual reproduction test (482 participants 60+ years)	Positive/no association	No association between fermented dairy intake and dementia risk (*P* = 0.26 for AOR) or cognitive performance scores (*P* > 0.05) Moderate cheese intake associated with lower dementia risk (AOR = 0.67, *P* = 0.05 for Q3 vs. Q1 intake)	Ylilauri et al. (28)

AOR, Adjusted odds ratio; FFQ, Food frequency questionnaire; HR, Hazard ratio; MMSE, Mini-Mental State Exam; Q1-Q4, Quintile 1–4 intake groups.

^a^Cohen's d calculated based on data given in the publication.

#### Total fermented dairy

Three cross-sectional studies considered fermented dairy products together as one group and the results were contradictory. The first found that higher intakes of fermented dairy products were associated with slighter better executive functioning (β = 0.06), which was also noted for skim dairy intake but not total dairy intake (22). Similarly, Tessier et al. (27) found higher fermented dairy intake was associated with slightly higher executive function scores (partial η^2^ = 0.002). In contrast, the third found that a high intake of fermented dairy products (Q4) increased the risk of lower MMSE scores (≤26) by 34% (26).

The difference in results between the studies is likely due to the type of cognitive assessment conducted. Specific benefits for the executive function domain were reported by Tessier et al. (27) and de Goeij et al. (22). However, Muñoz-Garach et al. (26) only screened global cognitive ability using the MMSE, which does not include executive function tasks, so that may account for why the same benefits of fermented dairy consumption were not observed. In addition, one prospective study examined the relationship between fermented dairy consumption and later-life cognition (28). It found that higher fermented dairy intake did not alter the risk of the development of dementia or improve cognitive performance in a range of cognitive domains, including executive function. This implies that fermented dairy consumption in mid-life did not have the same protective effect as that seen in the cross-sectional studies. Further research is required to confirm this.

#### Cheese

Cheese is a fermented dairy product that is high in SFA (~15%−25% depending on the variety), which as previously discussed has been implicated in an increased risk of developing cognitive impairment (35, 36). However, all six studies that specifically investigated the relationship between cheese intake and cognitive function in later life found a positive association.

Four of these were studies that evaluated cheese intake and cognition at the same point in time, which is not ideal. In a US-based study that separated cheese from milk consumption, it was found that an increased frequency of cheese intake reduced the risk of cognitive impairment by 32%, whereas no change in risk was associated with milk intake (31). In the previously discussed study by Park and Fulgoni (17) those who consumed cheese had a slightly better episodic memory than those who did not (Cohen's *d* = 0.12). This benefit was observed for those who consumed total dairy products, but no difference in cognition score was detected for those who did or did not consume milk. Whereas, the study by de Goeij et al. (22), which found no association between total dairy intake and any cognitive domain scores, and a positive association between skim milk intake and better executive function score, found that Dutch cheese intake (but not total cheese intake) was associated with a 33% reduced likelihood of low processing speed. Whereas, in the study by Tessier et al. (27), which found that those who consumed more dairy products performed better in tests measuring executive function and had no association between milk intake and cognitive test scores, cheese intake was also associated with slightly higher executive function (partial η^2^ = 0.001, *P* < 0.001). It is unclear why there were differences in which cognitive domain was affected between the studies, but it may be due to differences in the types of cognitive measures used.

In addition, two prospective cohort studies found positive associations between cheese intake and cognitive function. This includes a UK-based study in which those who consumed more cheese had better Fluid Intelligence Test scores (β = 0.207), which is an assessment of executive function problem-solving skills (39). In addition, the Finnish study which indicated that total dairy intake was not associated with dementia risk or cognitive test scores and milk intake was associated with lower verbal fluency test scores, found that moderate cheese intake (Q3) was associated with a 17% lower likelihood of developing dementia compared to low cheese intake (Q1) (28).

#### Yogurt

Yogurt is a fermented dairy product that is lower in SFA than cheese. Many yogurt products, particularly those sold in Asian countries, contain probiotic bacteria that have been selected for their health benefits. However, depending on the product, yogurt can contain added sugar which is likely to have a negative impact on cognitive function.

Three cross-sectional studies assessed the impact of yogurt consumption on cognition in later life and the results were conflicting. Kim and Yun (21) found that yogurt consumption decreased the odds of developing MCI by 74%. Tessier et al. (27) found that unlike cheese which was associated with better executive function, yogurt intake was associated with slightly better memory, however, the effect size was very small (partial η^2^ = 0.001). Whereas de Goeij et al. (22) did not find any association between total yogurt consumption and test performance for any cognitive domain tested (*P* > 0.05 for β).

The differences in results may be due to factors relating to the types of yogurts more popular in different countries. There may be differences in sugar content, the inclusion of beneficial probiotic bacteria or the frequency of consumption. For example, in South Korea where greater benefits were reported, liquid yogurt drinks that contain probiotic bacteria are more popular than in Western countries.

#### Buttermilk

Buttermilk is a fermented dairy product that is low in both SFA and sugar and therefore may be more beneficial than cheese and yogurt. However, buttermilk consumption was only analyzed separately in one study. As previously discussed, de Goeij et al. (22) found small improvements in executive function associated with fermented dairy consumption (β = 0.06), which was not explained by intakes of yogurt or cheese (*P* > 0.05 for β). However, buttermilk consumption was associated with increased executive function (β = 0.1), indicating that it is likely responsible for the observed benefits. Further research is needed to confirm this result.

## Experimental studies

Despite the growing observational evidence that regular, moderate consumption of dairy products is associated with maintained cognition during aging, few experimental studies have been conducted to confirm this association. There are no published studies assessing the effects of diet on cognitive aging where the dietary intervention is a whole dairy product (e.g., milk, yogurt, cheese).

All the experimental studies to date use dairy-derived peptides, such as those that are generated during the fermentation process, as the treatment. These six studies are summarized in Table 6 in the order in which they were published. They are discussed below in subsections based on the source of the peptides.

**Table 6:** Summary of experimental studies assessing the effects of dairy-derived treatments and cognitive function.

**Participants**	**Numbers**	**Design**	**Diet intervention**	**Cognitive tests**	**Outcome**	**Results**	**References**
50+ years with probably AD; living in Poland	105 (~1/3 men, 2/3 women)	Randomized, placebo-controlled, double-blind study	Tablets containing 0.1 mg colostrinin or placebo daily for 15 weeks, then 15 weeks where all participants received colostrinin	ADAS-cog, CGIC, IADL, MMSE	Positive	Colostrinin group had smaller changes in ADAS-cog, IADL and MMSE scores compared to placebo group (*P* < 0.05, SD not given so effect sizes unable to be calculated)	Bilikiewicz and Gaus (40)
45–65 years; self-diagnosed forgetfulness; living in Japan	98 (24 men, 64 women)	Randomized, placebo-controlled, double-blind study	Tablets containing 1 g of whey peptides (1.6 mg β-lactolin) or placebo daily for 12 weeks	Hamamatsu Higher Brain Function Scale, Rivermead Behavioral Memory Test, Stroop test, digit span, paced auditory serial addition test	Positive/no association	No difference in cognitive scores between treatment groups when all participants included (*P* > 0.05 for all test scores) Treatment improved verbal fluency for “a” score (Cohen's *d* = 0.73^a^) and reduced Stroop test step 3 errors (Cohen's *d* = 0.67^a^) compared to placebo after 6 weeks in high fatigue subgroup (n = 40)	Kita et al. (41)
50+ years; living in Japan	76	Two randomized, placebo-controlled, double-blind studies; with and without exercise invention	Tablets containing total of 3.4 mg lactotripeptide or placebo daily for 8 weeks	Stroop test	No association	No difference in Stroop test scores between treatment groups either with or without exercise (*P* = 0.087)	Hamasaki et al. (42)
50–75 years; self-diagnosed forgetfulness; living in Japan	104 (37 men, 67 women)	Randomized, placebo-controlled, double-blind study	Tablets containing 1 g of whey peptides (1.6 mg β-lactolin) or placebo daily for 12 weeks	WMS-R, clinical assessment for attention, standard verbal paired-associate test, recognition memory test for faces	Positive	Treatment improved visual paired-associates I (Cohen's *d* = 0.45^a^) and required time in visual cancelation task-Figure 2 (Cohen's *d* = 0.44^a^) compared to placebo	Kita et al. (43)
40+ years; living in Japan	268 (85 men, 183 women)	Randomized, placebo-controlled, double-blind study	Tablets containing 0.25 g of casein hydrolysate (0.05 mg MKP peptide) or placebo daily for 24 weeks	ADAS-cog, HDS-R, MoCA-J	Positive	Subgroup of 189 participants 65+ years had higher scores in the constructions and orientation subtest in the treatment group compared to placebo group (Cohen's *d* = 0.29 and 0.34^a^)	Yuda et al. (44)
45–65 years; self-diagnosed forgetfulness; living in Japan	30 (13 men, 17 women)	Randomized, placebo-controlled, double-blind study	Tablets containing 1 g of whey peptides (1.6 mg β-lactolin) or placebo daily for 6 weeks	Verbal fluency test, MCI screen, Cognitrax neurocognition index	Positive	Treatment increased verbal fluency for “a” score, but placebo did not (Cohen's *d* = 0.83^a^ for change in score after 6 weeks)	Kanatome et al. (45)

Green indicates a positive association between dairy-derived treatments and cognition and black indicates no association.

ADAS-cog, Alzheimer's Disease Assessment Scale - Cognition; CDIC, Clinical Global Impression of Change; HDS-R, Hierarchic Dementia Scale-Revised; IADL, Instrumental activities of daily living; MoCA-J, Montreal Cognitive Assessment-Japanese; MMSE, Mini-Mental State Exam; WMS-R, Wechsler Memory Scale-Revised.

^a^Cohen's d calculated based on data given in the publication.

### β-lactolin

Three of the experimental studies identified in the literature used a mixture of whey peptides as the dietary intervention (41, 43, 45). The whey peptides were produced by enzymatic digestion of whey protein, and the proposed active peptide, β-lactolin (glycine-threonine-tryptophan-tyrosine), made up 0.16% of the peptide mixture. β-lactolin is naturally produced by the fermentation of whey by the yeast *Penicillium candidum* and is found in camembert and blue cheeses. In all three studies, participants who received the whey peptide treatment showed greater improvement in specific cognitive domains compared to those who received the placebo.

Two of the studies had participants between 45 and 65 years who self-identified as having memory concerns. In the first, no differences in cognitive performance were noted between treatment groups when all participants were considered (41). However, when the data were separated based on fatigue level, those with high fatigue showed greater improvement in executive function tests 6 weeks after receiving the β-lactolin treatment compared to those who received the placebo (verbal fluency for “*a*” score, Cohen's *d* = 0.73; Stroop test step 3 errors, Cohen's *d* = 0.67). This result was confirmed in the second study where 6 weeks of β-lactolin treatment increased verbal fluency for “*a*” score, but the placebo treatment did not (Cohen's *d* = 0.83 for change in score) (45).

The third study using the same β-lactolin treatment used an older group of participants (50–75 years) and observed changes in different cognitive domains than in the slightly younger age group (43). β-lactolin treatment for 6 weeks improved visual paired-associates I (Cohen's *d* = 0.45) and required time in visual cancellation task-Figure 2 (Cohen's *d* = 0.44) compared to placebo, which are measures of visual memory and new learning, and visual selected attention, respectively. These results were supported by physiological measures that showed increased neural activity in the parietal area involved in audial attention and concentration.

### Proline-rich peptides

The other three experimental studies identified in the literature were similar in that they all used dairy-derived proline-rich peptides as the treatment. In one case the treatment was a proline-rich polypeptide mixture extracted from ovine colostrum, which was named colostrinin (40). In the other two studies, the proposed active ingredients were lactotripeptide, a combination of two proline-rich peptides (valine-proline-proline and isoleucine-proline-proline), and MKP peptide (methionine-lysine-proline), which are products of casein fermentation by lactic acid bacteria and casein hydrolysation, respectively (42, 44).

In the first study, where the participants were 50 years or older with probable AD, some positive effects were reported (40). Fifteen weeks of colostrinin treatment stabilized several global measures of cognition that continued to deteriorate over time in the placebo group. When the data were stratified, those with milder impairment had a more positive response to the treatment, than those with greater impairment. This implies that early intervention is more beneficial. However, only the means and not the standard deviations were reported so it was not possible to calculate the size of the effects.

The other two studies did not report cognitive benefits from the peptide intake. The primary aim of the study by Hamasaki et al. (42) was to assess the effect of lactotripeptide treatment on cerebral oxygenation in healthy participants over 50 years of age, but they also measured the participants' performance in the Stroop test. Oxygenation to the prefrontal cortex was increased in those who received lactotripeptide (both with and without exercise) compared to the placebo group; however, no differences in Stroop test scores were observed. In the other study in which participants were healthy individuals over 40 years of age, Yuda et al. (44) did not observe any differences between the cognitive scores of the MKP peptide and placebo treatment groups. When the data were stratified by age, those 65 years or older did not display any improvements in overall cognitive measures and only a slight improvement in two subtests (Constructions and Orientation Cohen's *d* = 0.29 and 0.34).

The lack of response to treatment in both of these studies may be because participants were cognitively healthy. Any improvements would be expected to be minor and therefore difficult to detect. Longer term studies where the effects of the dietary interventions in mid-life on cognitive outcomes in later-life are needed to assess the ability of the dairy-derived peptides to prevent cognitive impairment from occurring.

## Conclusions and future directions

This review of the literature provides evidence that moderate dairy consumption (approximately once per day) has a positive influence on cognitive function in later-life. This was most apparent in observational studies conducted in Asian countries where total dairy consumption was generally lower and a shift to adequate consumption was beneficial (15, 18, 19, 21, 24, 25). In Western countries, where average dairy consumption was higher, studies indicate that high consumption of dairy products negates the benefits observed by moderate consumption (23, 26–28). Collectively, the results of the observational studies imply that the relationship between dairy consumption and cognitive outcomes in later life is inverse U-shaped, with moderate consumption being the most beneficial.

Further analysis of the observational studies to determine which type(s) of dairy products were responsible for the benefits found that the outcomes from milk consumption were mixed. Like the results for total dairy consumption, cross-sectional studies indicated that increased milk consumption was more beneficial in Asian countries with lower average consumption (21) than in Western countries (17, 22, 27, 31). Three of the four prospective studies indicated that high milk consumption during mid-life was a risk factor for developing cognitive impairment in later life (28, 30, 33). In contrast, one retrospective study that evaluated childhood milk intake compared to later-life cognition found that early-life milk consumption had a protective effect (32). Based on these mixed results it is difficult to discern the effect of milk consumption on later-life cognition, however, it is unlikely that it is responsible for the benefits reported for moderate total dairy consumption.

Similar to the results for milk consumption, the effects of dairy lipid consumption on later-life cognition were inconsistent. Some studies found positive associations between dairy lipid intake (26), and some found no association (22, 27). The results may have been confounded by the inclusion of fermented dairy products in the analysis because studies that solely assessed low-fat vs. full-fat milk consumption found no difference in cognitive outcomes (22, 38). The consumption of high-fat dairy desserts was reported to be a risk factor for cognitive impairment (37); however, this was not necessarily due to the lipid intake and could instead be attributed to the high sugar content of the desserts. One particular study, which assessed the impact of the consumption of different types of dairy lipids found that PUFA and MUFA were associated with decreased risk of AD and MCI development, whereas, SFA intake was associated with increased risk (35, 36).

The final type of dairy product that was analyzed was fermented dairy products, such as cheese, yogurt and buttermilk. Of the three studies that assessed total fermented dairy intake, two found that higher consumption intake was associated with higher executive function scores (22, 27) but one found that higher intake was associated with lower cognitive scores (26). Despite cheese being the dairy product that is highest in SFA, it is also one that has the most consistent positive association with cognition during aging, with all six studies showing the benefits of cheese consumption (17, 22, 27, 28, 31, 39). Dutch cheese was shown to be particularly beneficial in the study by de Goeij et al. (22). This may be due to its higher vitamin K_2_ content compared to other cheeses (46), which may slow the development of AD (47). Perhaps, surprisingly, the results for yogurt were less consistent, with two studies finding positive associations (21, 27) and one showing no association (22). Although yogurt is lower in SFA it can be high in sugar content, depending on the product, which could account for the mixed results. The consumption of buttermilk, which is low in both SFA and sugar, was associated with improved executive function (22); however, this was only assessed in one study so further research is needed to confirm this.

Given that fermented dairy products such as cheese are high-value, their consumption is likely more common in wealthier individuals who may also have better access to healthcare, which could be a confounding factor for the results. All six studies that observed that cheese intake was beneficial for later-life cognition controlled for age, sex and education (a proxy for socioeconomic status) in their analyses. The majority also controlled for BMI, tobacco use and alcohol consumption, and some studies controlled for measures of physical activity, overall diet and various health conditions. Importantly, direct measures of socioeconomic status were controlled for in half of the studies including the Townsend Deprivation Index (39), poverty:income ratio (17), and income (27). This suggests that the benefits of cheese consumption are independent of socioeconomic status.

There were several differences in outcome measures between the observational studies which made it difficult to compare results, both for total dairy consumption and consumption of individual dairy products. For example, some studies focused on severe cognitive impairment and used measures of dementia or AD as the outcome measure, whereas others were concerned with more subtle changes and focused on indicators of MCI. In some studies, general measures of cognition were used, such as the MMSE, whereas in other studies more detailed assessment of various cognitive domains were carried out. This meant that some studies were unable to discern nuances in changes in cognition, which may account for why associations were not observed in some cases.

Another difference between the observational studies that made comparisons between results difficult was the variations in the type of study design used. For prospective studies, any positive relationships between dairy intake and cognition indicated a reduction in the risk of later-life cognitive impairment and therefore could be considered a prophylactic effect. However, with the cross-sectional studies, it is not possible to determine whether recorded dairy product intakes are indicative of life-long consumption and therefore any benefits can be attributed to a protective effect, or whether benefits associated with current dairy intake should be considered as a treatment effect. Several of the cross-sectional studies reported were baseline data from longitudinal studies that are currently underway (22, 25, 27). The future results of these studies will be critical to the understanding of the relationship between life-long diet and later-life cognitive outcomes.

To truly prove that moderate consumption of dairy products is beneficial for protecting against or treating cognitive impairment in later life, randomized, double-blinded, placebo-controlled dietary intervention experiments are needed. To assess the protective effects, a long-term dietary intervention study where participants are given low, moderate or high amounts of dairy daily would be needed. This would be technically and ethically challenging because a time frame of at least 10 years would be needed due to the slow progression of cognitive impairment with aging. It would be more feasible to assess the benefits of increased dairy consumption as a treatment for those already experiencing cognitive impairment. For example, participants with low baseline intakes of dairy and cognitive impairment could be treated with a moderate intake of dairy daily.

To date, human food intervention studies have only used dairy-derived peptides, not whole dairy products. This is not surprising given these peptides are products of dairy fermentation, and fermented dairy products are the most likely to be responsible for the observed benefits of dairy consumption. Of the six reported experimental studies, in the four studies in which participants had reported cognitive impairments the dairy peptide intervention resulted in improvements in cognitive scores (40, 41, 43, 45); whereas in the two studies where the participants were cognitively healthy, no improvements in cognition were reported (42, 44). Collectively, these results indicate that dairy-derived peptides could be an effective treatment for early-stage cognitive impairment associated with aging.

Another area for future research is understanding the mechanisms by which dairy product consumption alters cognition. For the majority of the observational studies the statistical modeling controls for confounding factors including diet quality and overall intake, therefore, it is unlikely that the benefits of dairy are purely due to increased nutrition. It is possible that the benefits occur indirectly via modulation of the gut microbiota and/or reduction in gut permeability, which in turn decreases neuroinflammation. Future studies should combine dietary assessment and cognitive testing alongside physiological measures, such as gut microbiota composition, gut permeability and blood biomarkers of inflammation, to elucidate the mechanisms of action.

Future studies could also investigate the role of dairy lipids that have been metabolized by bacteria in maintaining cognition during aging. The lipid profiles of fermented dairy products are remarkably different to milk (48). For example, triglycerides are twice as abundant in yogurt and 10 times more abundant in cheese compared to milk, indicating that medium-chain fatty acids are broken down by the bacteria. The lipid profiles of cheese are also distinct from those of yogurt which may contribute to the difference in their reported benefits.

The narrative review approach taken here has both strengths and limitations. This review brings together information from a range of study types including cross-sectional, retrospective and prospective observational studies, as well as experimental food intervention studies. This has the strength of giving a broad overview of the field and synthesis of knowledge to inform readers. A limitation of the approach is that due to the large range of study types reviewed a meta-analysis was not possible, so the conclusions may be considered less objective. To attempt to reduce any biases, predefined search methods and inclusion/exclusion criteria were used to select studies to be reviewed.

In conclusion, this review indicates that dairy consumption, particularly of fermented products, reduces the risk of cognitive impairment in later life. It is unclear whether this is due to a protective effect of lifelong consumption, or a treatment effect when cognitive impairment occurs. Further experimental studies with whole dairy product dietary interventions, particularly with fermented dairy products, are needed to determine whether the regular, moderate consumption (e.g., 1–2 servings per day) of these foods should be recommended to maximize the likelihood of healthy cognitive aging.

## Author contributions

RA: Writing – original draft, Methodology, Funding acquisition, Formal analysis, Data curation, Conceptualization. FA: Writing – review & editing.

## References

[1] World Health Organisation. Ageing and Health. (2021). Available online at: https://www.who.int/news-room/fact-sheets/detail/ageing-and-health (accessed September 24, 2022).

[2] Salthouse TA. Selective review of cognitive aging. J Int Neuropsychol Soc. (2010) 16:754–60. 10.1017/S1355617710000706 20673381 PMC3637655

[3] Nordahl CW Ranganath C Yonelinas AP DeCarli C Reed BR Jagust WJ. Different mechanisms of episodic memory failure in mild cognitive impairment. Neuropsychologia. (2005) 43:1688–97. 10.1016/j.neuropsychologia.2005.01.003 16009250 PMC3771323

[4] Nordestgaard LT Christoffersen M Frikke-Schmidt R. Shared risk factors between dementia and atherosclerotic cardiovascular disease. Int J Mol Sci. (2022) 23:9777. 10.3390/ijms23179777 36077172 PMC9456552

[5] Soenen S Rayner CK Jones KL Horowitz M. The ageing gastrointestinal tract. Curr Opin Clin Nutr Metab Care. (2016) 19:12–8. 10.1097/MCO.0000000000000238 26560524

[6] Fard MT Savage KM Stough CK. Peripheral inflammation marker relationships to cognition in healthy older adults – a systematic review. Psychoneuroendocrinology. (2022) 144:105870. 10.1016/j.psyneuen.2022.105870 35908534

[7] Nichols E Szoeke CEI Vollset SE Abbasi N Abd-Allah F Abdela J. Global, regional, and national burden of Alzheimer's disease and other dementias, 1990-2016: a systematic analysis for the Global Burden of Disease Study 2016. Lancet Neurol. (2019) 18:88–106. 10.1016/S1474-4422(18)30403-4 30497964 PMC6291454

[8] ChartBin Statistics Collector Team. Current Worldwide Total Milk Consumption per capita. (2011). Available online at: http://chartsbin.com/view/1491 (accessed April 6, 2023).

[9] Kim J Yu A Choi BY Nam JH Kim MK Oh DH. Dietary patterns and cognitive function in Korean older adults. Eur J Nutr. (2015) 54:309–18. 10.1007/s00394-014-0713-0 24842708

[10] Shin D Lee KW Kim M-H Kim HJ An YS Chung H-K. Identifying dietary patterns associated with mild cognitive impairment in older Korean adults using reduced rank regression. Int J Environ Res Public Health. (2018) 15:100. 10.3390/ijerph15010100 29315276 PMC5800199

[11] Chuang S-Y Lo Y-L Wu S-Y Wang P-N Pan W-H. Dietary patterns and foods associated with cognitive function in taiwanese older adults: the cross-sectional and longitudinal studies. J Am Med Dir Assoc. (2019) 20:544. 10.1016/j.jamda.2018.10.017 30630727

[12] LuYGweeXChuaDQLeeTSLimWSChongMS. Nutritional status and risks of cognitive decline and incident neurocognitive disorders: singapore longitudinal ageing studies. J Nutr Health Aging. (2021) 25:660–7. 10.1007/s12603-021-1603-933949634

[13] Su X Zhang J Wang W Ni C Hu S Shao P. Dietary patterns and risk of mild cognitive impairment among Chinese elderly: a cross-sectional study. PLoS ONE. (2020) 15:e0235974. 10.1371/journal.pone.0235974 32658926 PMC7357755

[14] Xu X Parker D Shi Z Byles J Hall J Hickman L. Dietary pattern, hypertension and cognitive function in an older population: 10-year longitudinal survey. Front Public Health. (2018) 6:201. 10.3389/fpubh.2018.00201 30079333 PMC6062638

[15] Lee L Kang SA Lee HO Lee BH Park JS Kim JH. Relationships between dietary intake and cognitive function level in Korean elderly people. Public Health. (2001) 115:133–8. 10.1016/S0033-3506(01)00432-2 11406779

[16] Chen RC Chang YH Lee MS Wahlqvist ML. Dietary quality may enhance survival related to cognitive impairment in Taiwanese elderly. Food Nutr Res. (2011) 55. 10.3402/fnr.v55i0.7387 22046146 PMC3205824

[17] Park KM Fulgoni VL. The association between dairy product consumption and cognitive function in the National Health and Nutrition Examination Survey. Br J Nutr. (2013) 109:1135–42. 10.1017/S0007114512002905 23168329

[18] Otsuka R Kato Y Nishita Y Tange C Nakamoto M Tomida M. Cereal intake increases and dairy products decrease risk of cognitive decline among elderly female Japanese. J Prev Alzheimers Dis. (2014) 1:160–7. 10.14283/jpad.2014.29 29251743

[19] Ozawa M Ohara T Ninomiya T Hata J Yoshida D Mukai N. Milk and dairy consumption and risk of dementia in an elderly japanese population: the Hisayama Study. J Am Geriatr Soc. (2014) 62:1224–30. 10.1111/jgs.12887 24916840

[20] PilleronSDesportJCJésusPMbelessoPNdamba-BandzouziBDartiguesJF. Diet, alcohol consumption and cognitive disorders in Central Africa: a study from the EPIDEMCA program. J Nutr Health Aging. (2015) 19:657–67. 10.1007/s12603-015-0487-y26054502

[21] Kim KY Yun J-M. Association between diets and mild cognitive impairment in adults aged 50 years or older. Nutr Res Pract. (2018) 12:415–25. 10.4162/nrp.2018.12.5.415 30323909 PMC6172167

[22] de Goeij LC van de Rest O Feskens EJM de Groot L Brouwer-Brolsma EM. Associations between the intake of different types of dairy and cognitive performance in Dutch Older adults: the B-PROOF study. Nutrients. (2020) 12:468. 10.3390/nu12020468 32069791 PMC7071379

[23] LiYLiSWangWZhangDF. Association between dietary protein intake and cognitive function in adults aged 60 years and older. J Nutr Health Aging. (2020) 24:223–9. 10.1007/s12603-020-1317-432003415

[24] Talaei M Feng L Yuan J-M Pan A Koh W-P. Dairy, soy, and calcium consumption and risk of cognitive impairment: the Singapore Chinese Health Study. Eur J Nutr. (2020) 59:1541–52. 10.1007/s00394-019-02010-8 31161350 PMC6888923

[25] Huang Q Jia X Zhang J Huang F Wang H Zhang B. Diet-cognition associations differ in mild cognitive impairment subtypes. Nutrients. (2021) 13:1341. 10.3390/nu13041341 33920687 PMC8073801

[26] Muñoz-Garach A Cornejo-Pareja I Martínez-González M Bulló M Corella D Castañer O. Milk and dairy products intake is related to cognitive impairment at baseline in predimed plus trial. Mol Nutr Food Res. (2021) 65:e2000728. 10.1002/mnfr.202000728 33471961

[27] Tessier A-J Presse N Rahme E Ferland G Bherer L Chevalier S. Milk, yogurt, and cheese intake is positively associated with cognitive executive functions in older adults of the Canadian longitudinal study on aging. J Gerontol A Biol Sci Med Sci. (2021) 76:2223–31. 10.1093/gerona/glab165 34115853

[28] Ylilauri MPT Hantunen S Lonnroos E Salonen JT Tuomainen T-P Virtanen JK. Associations of dairy, meat, and fish intakes with risk of incident dementia and with cognitive performance: the Kuopio Ischaemic Heart Disease Risk Factor Study (KIHD). Eur J Nutr. (2022) 61:2531–42. 10.1007/s00394-022-02834-x 35217900 PMC9279192

[29] Yamada M Kasagi F Sasaki H Masunari N Mimori Y Suzuki G. Association between dementia and midlife risk factors: the radiation effects research foundation adult health study. J Am Geriatr Soc. (2003) 51:410–4. 10.1046/j.1532-5415.2003.51117.x 12588587

[30] Almeida OP Norman P Hankey G Jamrozik K Flicker L. Successful mental health aging: results from a longitudinal study of older Australian men. Am J Geriatr Psychiatry. (2006) 14:27–35. 10.1097/01.JGP.0000192486.20308.42 16407579

[31] Rahman A Baker PS Allman RM Zamrini E. Dietary factors and cognitive impairment in community-dwelling elderly. J Nut Health Aging. (2007) 11:49–54. 17315080

[32] Zhang ZX Plassman BL Xu Q Zahner GE Wu B Gai MY. Lifespan influences on mid- to late-life cognitive function in a Chinese birth cohort. Neurology. (2009) 73:186–94. 10.1212/WNL.0b013e3181ae7c90 19620606 PMC2843580

[33] Petruski-Ivleva N Kucharska-Newton A Palta P Couper D Meyer K Graff M. Milk intake at midlife and cognitive decline over 20 years the atherosclerosis risk in communities (ARIC) study. Nutrients. (2017) 9:1134. 10.3390/nu9101134 29039795 PMC5691750

[34] Schipper L van Dijk G van der Beek EM. Milk lipid composition and structure; the relevance for infant brain development. OCL Oilseeds Fats Crops Lipids. (2020) 27:5. 10.1051/ocl/2020001

[35] Laitinen MH Ngandu T Rovio S Helkala EL Uusitalo U Viitanen M. Fat intake at midlife and risk of dementia and Alzheimer's disease: a population-based study. Dement Geriatr Cogn Disord. (2006) 22:99–107. 10.1159/000093478 16710090

[36] Eskelinen MH Ngandu T Helkala E-L Tuomilehto J Nissinen A Soininen H. Fat intake at midlife and cognitive impairment later in life: a population-based CAIDE study. Int J Geriatr Psychiatry. (2008) 23:741–7. 10.1002/gps.1969 18188871

[37] Vercambre MN Boutron-Ruault MC Ritchie K Clavel-Chapelon F Berr C. Long-term association of food and nutrient intakes with cognitive and functional decline: a 13-year follow-up study of elderly French women. Br J Nutr. (2009) 102:419–27. 10.1017/S0007114508201959 19203415 PMC2891709

[38] Franca VF Azzolini T Pissaia E Bortoloti DS Signorini T Dalla Costa L. Diet, epidemiological factors and cognitive impairment: a cross-sectional study in the elderly population. Braz Arch Biol Technol. (2018) 61:e18180225. 10.1590/1678-4324-2018180225

[39] Klinedinst BS Le ST Larsen B Pappas C Hoth NJ Pollpeter A. Genetic factors of Alzheimer's disease modulate how diet is associated with long-term cognitive trajectories: a UK Biobank Study. J Alzheimers Dis. (2020) 78:1245–57. 10.3233/JAD-201058 33252089 PMC7895545

[40] Bilikiewicz A Gaus W. Colostrinin1 (a naturally occuring, proline-rich, polypeptide mixture) in the treatment of Alzheimer's disease. J Alzheimers Dis. (2004) 6:17–26. 10.3233/JAD-2004-6103 15004324

[41] Kita M Obara K Kondo S Umeda S Ano Y. Effect of supplementation of a whey peptide rich in tryptophan-tyrosine-related peptides on cognitive performance in healthy adults: a randomized, double-blind, placebo-controlled study. Nutrients. (2018) 10:899. 10.3390/nu10070899 30011836 PMC6073406

[42] Hamasaki A Akazawa N Yoshikawa T Myoenzono K Tanahashi K Sawano Y. Combined effects of lactotripeptide and aerobic exercise on cognitive function and cerebral oxygenation in middle-aged and older adults. Am J Clin Nutr. (2019) 109:353–60. 10.1093/ajcn/nqy235 30624594

[43] Kita M Kobayashi K Obara K Koikeda T Umeda S Ano Y. Supplementation with whey peptide rich in beta-lactolin improves cognitive performance in healthy older adults: a randomized, double-blind, placebo-controlled study. Front Neurosci. (2019) 13:399. 10.3389/fnins.2019.00399 31068787 PMC6491855

[44] Yuda N Tanaka M Yamauchi K Abe F Kakiuchi I Kiyosawa K. Effect of the casein-derived peptide met-lys-pro on cognitive function in community-dwelling adults without dementia: a randomized, double-blind, placebo-controlled trial. Clin Interv Aging. (2020) 15:743–54. 10.2147/CIA.S253116 32546992 PMC7266326

[45] Kanatome A Ano Y Shinagawa K Ide Y Shibata M Umeda S. β-lactolin enhances neural activity, indicated by event-related P300 amplitude, in healthy adults: a randomized controlled trial. J Alzheimers Dis. (2021). 81:787–96. 10.3233/JAD-201413 33814437 PMC8203246

[46] Vermeer C Raes J Van 't Hoofd C Knapen MHJ Xanthoulea S. Menaquinone content of cheese. Nutrients. (2018) 10:446. 10.3390/nu10040446 29617314 PMC5946231

[47] Popescu A German M. Vitamin K2 holds promise for Alzheimer's prevention and treatment. Nutrients. (2021) 13. 10.3390/nu13072206 34199021 PMC8308377

[48] Furse S Torres AG Koulman A. Fermentation of milk into yoghurt and cheese leads to contrasting lipid and glyceride profiles. Nutrients. (2019) 11:2178. 10.3390/nu11092178 31514309 PMC6770487

